# Efficacy and safety of vesicular monoamine transporter 2 inhibitors for Huntington’s disease chorea based on network meta-analysis

**DOI:** 10.3389/fphar.2025.1637577

**Published:** 2025-09-24

**Authors:** Jing Huang, Fei-Fei Chen, Si-Yuan Wen, Pan Tao, Chi Meng, Wei Chen, Chang-Qing Zhou

**Affiliations:** Department of Neurology, Bishan Hospital of Chongqing Medical University, Chongqing, China

**Keywords:** huntington’s disease, chorea, VMAT2 inhibitors, tetrabenazine, deutetrabenazine, valbenazine, network meta-analysis

## Abstract

**Objective:**

A Bayesian network meta-analysis was conducted to evaluate the efficacy, tolerability, and safety of vesicular monoamine transporter 2 (VMAT2) inhibitors in Huntington’s disease chorea.

**Methods:**

The MEDLINE, Embase, and Cochrane Central Register of Controlled Trials were searched from January 1970 to January 2025 for eligible randomized controlled trials. Three VMAT2 inhibitors, including tetrabenazine, deutetrabenazine and valbenazine were investigated. The network meta-analysis was conducted based on a Bayesian framework using a fixed-effects model. As there were no closed loops in the network plot, comparisons of these interventions were directly formed by a consistency model.

**Results:**

Three randomized controlled trials (n = 299 patients) were included in the analysis. The surface under the cumulative ranking curve indicated that tetrabenazine was associated with the greatest improvement in the Unified Huntington’s Disease Rating Scale Total Maximal Chorea score (0.878), followed by valbenazine (0.700) and deutetrabenazine (0.422). Meanwhile, in the Unified Huntington’s Disease Rating Scale Total Motor score, valbenazine ranked highest, with a score of 0.781. Deutetrabenazine ranked highest in terms of overall withdrawals (0.800) and adverse events (AEs) (0.688), while valbenazine ranked first in withdrawals due to AEs (0.735), serious adverse events (0.807), as well as in reducing both suicide (0.683) and suicidal ideation (0.748).

**Conclusion:**

This study suggests that three VMAT2 inhibitors are effective in ameliorating chorea symptoms in patients with Huntington’s disease. Tetrabenazine is the most effective in controlling chorea, whereas valbenazine may be the optimal choice for patients with comorbid psychiatric symptoms.

**Systematic Review Registration:**

https://www.crd.york.ac.uk/PROSPERO/view/CRD420251012431, identifier CRD420251012431.

## 1 Introduction

Huntington’s disease (HD) is a hereditary neurodegenerative disorder characterized by progressive motor, cognitive, and psychiatric dysfunction (Stoker et al., 2022). Chorea is the hallmark feature of HD. These involuntary, purposeless movements are particularly debilitating, severely impacting patients’ quality of life and functional independence (Thorley et al., 2018).

At present, therapeutic strategies for chorea focus on modulating the dopaminergic system, primarily through the vesicular monoamine transporter 2 (VMAT2) inhibitors, which reduces dopamine release and alleviates chorea symptoms (Gibson and Claassen, 2021). Tetrabenazine was the first VMAT2 inhibitor approved by the U.S. Food and Drug Administration for the treatment of chorea in HD (Huntington Study Group, 2006). Tetrabenazine is effective in reducing chorea, but its use is limited by side effects like depression and somnolence. Additionally, three daily administrations of tetrabenazine further restrict patient adherence. To address these limitations, next-generation VMAT2 inhibitors, including deutetrabenazine and valbenazine, have been developed (Frank et al., 2016; Stimming et al., 2023). Deutetrabenazine, a deuterated form of tetrabenazine, exhibits a longer half-life and reduced metabolic variability, allowing for twice-daily dosing and improved tolerability. Valbenazine, a highly selective VMAT2 inhibitor, is metabolized into a single active metabolite, [+]-α-dihydrotetrabenazine, which exhibits the strongest affinity for VMAT2, thereby providing enhanced stability and predictability in its therapeutic effects. Moreover, a significant advantage of valbenazine lies in its once-daily dosing regimen. This streamlined approach eliminates the need for complex dose titration, thereby increasing treatment adherence and convenience, particularly for patients requiring long-term therapy (Neurocrine Biosciences, 2022; Harriott et al., 2018).

Over the past few decades, only a limited number of randomized controlled trials (RCTs) have been conducted to evaluate VMAT2 inhibitors in Huntington’s disease (HD), and no head-to-head RCT has directly compared tetrabenazine, deutetrabenazine, and valbenazine. Current international guidelines (Bachoud-Lévi et al., 2019) acknowledge deutetrabenazine as a clinically viable alternative to tetrabenazine, but provide no specific recommendations for valbenazine. To our knowledge, no previous network meta-analysis (NMA) or other strong evidence has directly demonstrated that a given active VMAT2 inhibitor is more potent than another in HD. To address this issue, we conducted a Bayesian network meta-analysis to assess the efficacy, tolerability, and safety of tetrabenazine, deutetrabenazine, and valbenazine in the HD, aiming to provide evidence-based insights to guide clinical decision-making.

## 2 Methods

Our analysis was guided by principles of the PRISMA (Preferred Reporting Items for Systematic Reviews and Meta-Analyses) Extension Statement (Hutton et al., 2015).

### 2.1 Search strategy and data extraction

Using a search strategy (Supplementary Appendix S1), we searched the literature in the MEDLINE (PubMed interface), Embase and Cochrane Controlled Register of Controlled Trials from 1 January 1970, to 1 January 2025. Meanwhile, we also conducted a manual search of original studies included in published meta-analyses, systematic reviews, and ongoing or unpublished trials and abstracts. After removing duplicates, two reviewers (J.H. and F.-F.C.) independently screened titles and abstracts and extracted data on study characteristics, including the first/corresponding author, study design, publication year, country, sample size, interventions, and outcome measures. Discrepancies were resolved through discussion or, if no consensus could be reached, by an adjudicator (C.-Q.Z.). Missing data were obtained by contacting the original authors whenever possible.

### 2.2 Inclusion and exclusion criteria

The search strategy was based on the PICOS principle (P: population/patient, I: intervention, C: control/comparison, O: outcome, S: study design) (Lu et al., 2023). In terms of patients, the following criteria were included: patients diagnosed with Huntington’s disease (HD) manifesting chorea, with no restrictions applied to age, race, or gender. In terms of interventions, the following criteria were considered: the group administered with vesicular monoamine transporter type 2 (VMAT2) inhibitors (including tetrabenazine, deutetrabenazine, and valbenazine) was considered the treatment group. In terms of comparators, the control group included any active comparator (other VMAT2 inhibitors) or placebo. In terms of study design, the following criteria were considered: we included randomized controlled trials (RCTs). In terms of outcomes, the following criteria were considered: the outcome indicators are change in the Unified Huntington’s Disease Rating Scale (UHDRS) Total Maximal Chorea (TMC) score, change in UHDRS Total Motor Score, Patient Global Impression of Change (PGI-C), Clinical Global Impression of Change (CGI-C), overall withdrawal rates, withdrawals due to adverse events (AEs), dose reduction due to intolerance, AEs with a higher probability of occurrence (including somnolence, fatigue, fall, diarrhea, depression and insomnia), serious adverse events (SAEs), suicide and suicide ideation, and change in Barnes Akathisia Scale (Global and Total) (BARS-G and BARS-T).

The exclusion criteria included the following: studies including HD patients with severe psychiatric disorders, studies including HD patients with a history of epilepsy or convulsions, studies including HD patients with clinically relevant hepatic, renal, or cardiac disorders that could interfere with the safety or tolerability of VMAT2 inhibitors, data that were missing or could not be extracted, non-RCTs, duplicated publications, conference reports, systematic reviews, or meta-analyses, and studies that demonstrated a high risk of bias.

### 2.3 Outcomes

The study assessed efficacy, tolerability, and safety outcomes. The primary efficacy endpoint was the change in the UHDRS TMC score, defined as the difference between baseline and the average value during the maintenance therapy period. For studies involving tetrabenazine and deutetrabenazine, the average was calculated over week 9 and 12, whereas for valbenazine studies, the average was derived from week 10 and 12. Secondary efficacy endpoints included: The CGI-C and PGI-C responses, defined as the proportion of participants achieving a rating of “much improved” or “very much improved” at week 12. Additional exploratory endpoints included: The change in UHDRS Total motor score from baseline to week 12. Tolerability and safety outcomes encompassed: (1) Overall withdrawals, withdrawals due to adverse events, and dosage reductions for any reason. (2) SAEs and AEs. (3) Suicidal ideation and behaviors. (4) The change in the ESS score, the BARS-G score, and the BARS-T score from baseline to week 12.

### 2.4 Risk of bias assessment

Risk of bias was independently assessed by two reviewers (J.H. and F.-F.C.) who used the Cochrane Risk of Bias Tool for randomized clinical trials (RoB 2) (Sterne et al., 2019). The certainty of the evidence was assessed using the CINeMA framework (Confidence in Network Meta-Analysis; accessible at https://cinema.ispm.unibe.ch/), which evaluates confidence in NMA estimates across six key domains: within-study bias, reporting bias, indirectness, imprecision, heterogeneity, and incoherence (Nikolakopoulou et al., 2020; Papakonstantinou et al., 2020). Any discrepancies in the CINeMA assessments were resolved through consensus discussions among the reviewers.

### 2.5 Statistical analysis

Various intervention measures were compared using Bayesian NMA in accordance with established Bayesian modeling guidelines (Dias et al., 2013). NMA is an analytical approach that synthesizes both direct and indirect evidence, thereby overcoming the constraints of pairwise comparisons and facilitating the simultaneous evaluation of multiple interventions. Bayesian models enable more efficient data integration and yield more reliable ranking outcomes. All Bayesian analyses were executed in R software (version 4.2.3) using the gemtc package, with a Gibbs sampler employed for Markov chain Monte Carlo simulations. The analysis utilized four simulation chains, with 2,0000 adaptation iterations and 50,000 simulation iterations. In cases where the data were particularly sparse, the number of adaptation iterations was increased to 50,000 and simulation iterations to 200,000 to ensure robust model convergence. Additionally, Fisher’s exact test was employed to determine whether observed differences were statistically significant when data sparsity posed challenges for Bayesian estimation. For continuous outcomes, the mean difference (MD) was utilized as the effect measure, with uncertainty quantified using 95% credible intervals (CrIs). For binary outcomes, the odds ratio (OR) and 95% CrI were applied. The Surface Under the Cumulative Ranking Curve (SUCRA) was employed to assess the ranking probabilities of different medications across various outcomes. For reverse-scored outcome measures, a data transformation was applied in the original R code so that higher SUCRA values consistently indicate more favorable results for patients with HD across all efficacy, safety, and tolerability endpoints. In cases where data for a specific treatment group were missing, comparisons were limited to the available drugs and placebo. In the original studies of deutetrabenazine, due to data unavailability, the number of participants experiencing AEs was approximated by the frequency of adverse events, which did not affect the overall conclusions of the AEs.

## 3 Results

### 3.1 Search results and study characteristics

Our search yielded 498 potential literature citations, of which three RCTs met the inclusion criteria (1 tetrabenazine vs. placebo, 1 deutetrabenazine vs. placebo, 1 valbenazine vs. placebo) (Huntington Study Group, 2006; Huntington Study Group et al., 2016; Stimming et al., 2023). And a total of 299 HD patients were included in present NMA (Supplementary Figure S1). Baseline characteristics of participants are summarized in Table 1 [mean age of approximately 52 years and a higher proportion (approximately 60%) of women than men]. Trials were generally well-balanced with respect to patient baseline characteristics and the Cochrane risk of bias assessment for included studies is presented in Supplementary Figure S1.

**TABLE 1:** Baseline characteristics of included studies in the network meta-analysis.

Trials (years)	Country	Study arm (n)	Mean age, years	Female sex, %	White, %	CAGn	OTD, weeks	Dosage, mg	UHDRS TMC score
TETRA-HD (2006)	United States of America	TBZ (54) PBO (30)	49.4 48.8	61 63	93 97	44.9 44.3	12	12.5–100 /	14.7 15.2
First-HD (2016)	United States of America Canada	DTBZ (45) PBO (45)	55.4 52.1	51 38	100 84	43.4 44.3	12	6–48 /	12.1 13.2
KINECT-HD (2023)	United States of America Canada	VBZ (64) PBO (61)	54.1 53.3	52 57	94 98	43.5 43.3	12	40–80 /	12.2 12.1

Abbreviations: CAGn, trinucleotide repeat length for expanded HD, allele; DTBZ, deutetrabenazine; OTD, overall treatment duration; PBO, placebo; TBZ, tetrabenazine; UHDRS TMC, Unified Huntington’s Disease Rating Scale Total Maximal Chorea; VBZ, valbenazine.

### 3.2 Quality assessment

**FIGURE 1:**
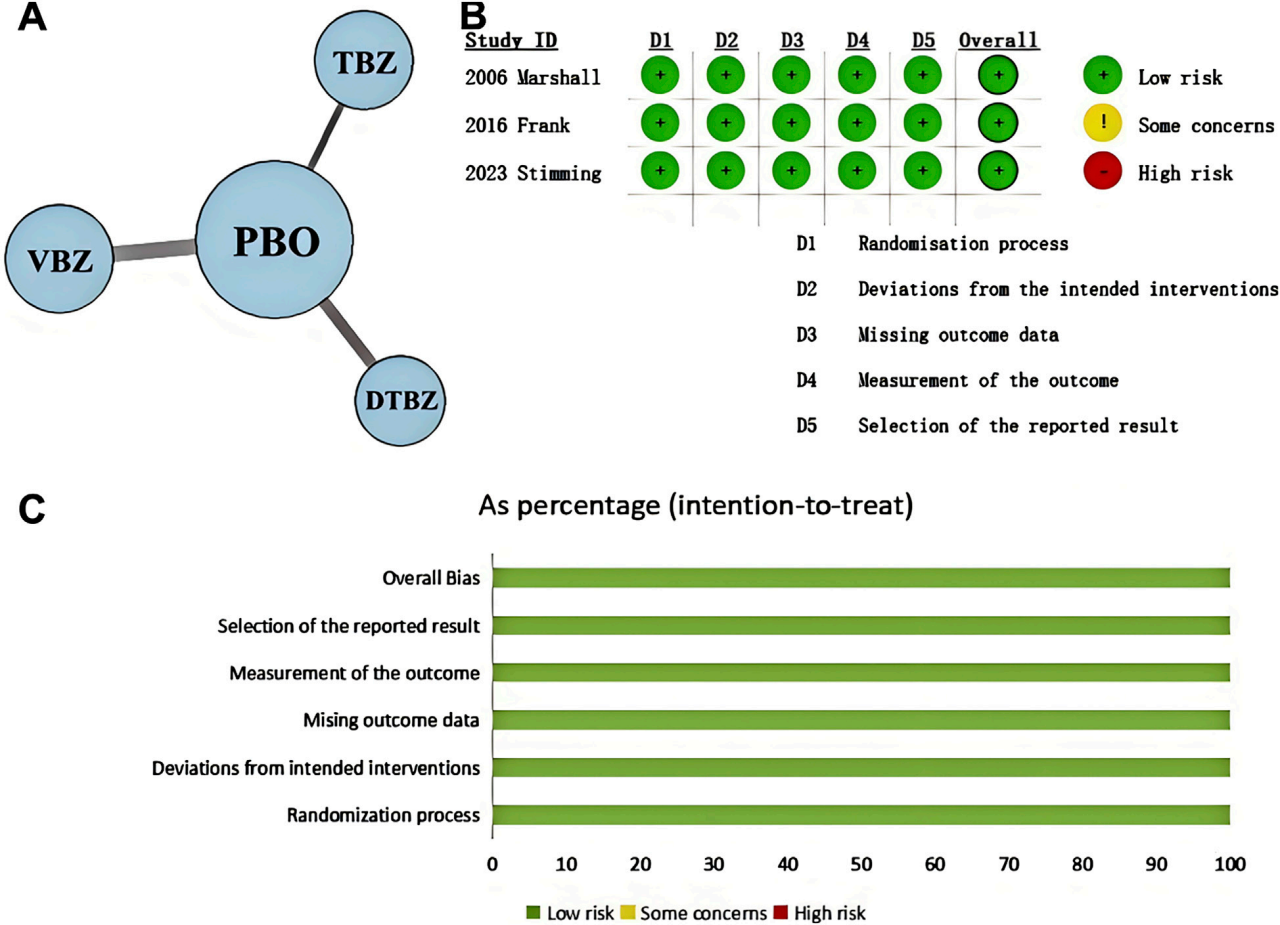
**(A)** Network plot shows patient counts per treatment arm for outcomes, with line width representing sample size for direct comparisons. (KINECT-HD included two incomplete subjects in safety/tolerability analysis.) **(B)** Cochrane system bias evaluations for each of the included publications. **(C)** Risk of bias graph. DTBZ, deutetrabenazine; PBO, placebo; TBZ, tetrabenazine; VBZ, valbenazine.

To clearly represent the comparisons within the NMA, we have created a network plot (Figure 1). Given that all indicators in the I^2^ heterogeneity test were less than 50%, and no significant differences were observed when compared to the random effects model (model fit details in Supplementary Table S1), the fixed effects model was selected for this NMA. The potential scale reduction factors (PSRFs) were all close to one, suggesting that the number of iterative simulations performed was sufficient to achieve good convergence, as shown by the PSRFs, trace plot, and density plot (Supplementary Figure S2). The risk of bias assessment results are presented in Figure 1. Confidence in the evidence based on the CINeMA framework is summarized in Supplementary Table S4, though formal evaluation of imprecision, heterogeneity, and incoherence was not feasible due to constraints inherent in the analytical methodology and the limited number of included studies.

### 3.3 Efficacy outcomes

#### 3.3.1 UHDRS Total Maximal Chorea score

**FIGURE 2:**
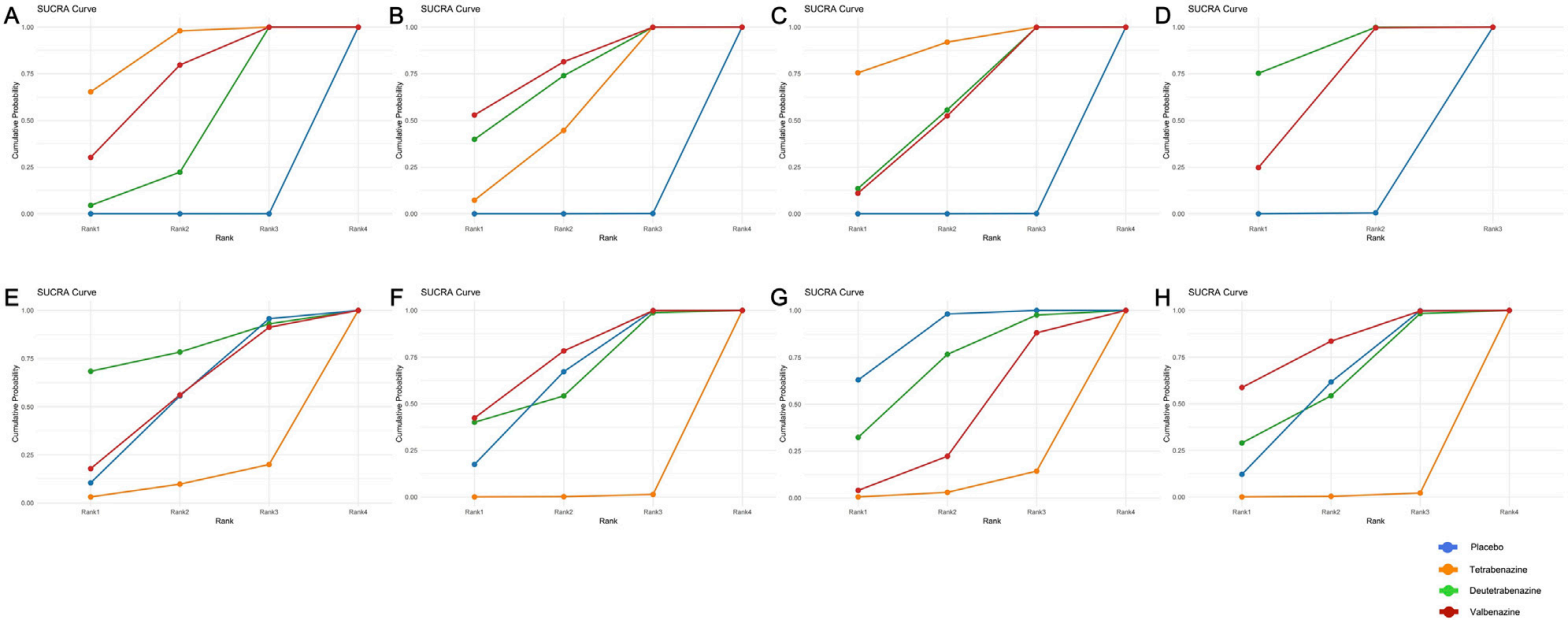
Surface under the cumulative ranking curve of available competing interventions for main outcomes. **(A)** = UHDRS TMC, **(B)** = UHDRS Total motor, **(C)** = CGI-C response, **(D)** = PGI-C response, **(E)** = Overall withdrawals, **(F)** = Withdrawals due to AEs, **(G)** = AEs, **(H)** = SAE.

Compared with placebo, tetrabenazine, valbenazine, and deutetrabenazine exhibited increased efficacy (MD = −3.50, 95% CrI = −3.78 to −3.22; MD = −3.20, 95% CrI = −4.37 to −2.03; MD = −2.50, 95% CrI = −3.71 to −1.30, respectively) (Table 2). The results of this analysis indicated that there were no statistically significant differences among the three interventions. The SUCRAs indicate a ranking of the three potential best treatments as tetrabenazine (0.878), valbenazine (0.700), and deutetrabenazine (0.422) (Figure 2A).

**TABLE 2:** Fixed-effects model measures for interventions evaluated in the included studies have been represented by the mean difference.

Outcomes measured	Network meta-analysis results (MD; 95% CI)						SUCRA			
	TBZ vs. PBO	DTBZ vs. PBO	VBZ vs. PBO	TBZ vs. DTBZ	TBZ vs. VBZ	DTBZ vs. VBZ	TBZ	DTBZ	VBZ	PBO	
UHDRS TMC	**−3.50 (-3.78,-3.22)**	**−2.50 (-3.71,-1.30)**	**−3.20 (-4.37,-2.03)**	−1.00 (−2.23,0.24)	−0.30 (−1.51,0.90)	0.70 (−0.98,2.37)	**0.878**	0.422	0.700	0.000	
UHDRS Total motor	**−3.30 (-3.91,-2.69)**	**−4.00 (-1.61,-1.01)**	**−4.30 (-6.81,-1.78)**	0.70 (−1.85,3.24)	1.00 (−1.59,3.59)	0.30 (−3.21,3.82)	0.506	0.712	**0.781**	0.000	
ESS	**−1.80 (1.55,2.05)**	−0.30 (−2.6,2.0)	/	**−2.10 (0.77,3.43)**	/	/	0.000	**0.835**	/	0.664	
BARS-G	/	−0.20 (−0.48,0.08)	**0.30 (0.06,0.54)**	/	/	**−0.50 (-0.87,-0.13)**	/	**0.958**	0.005	0.537	
BARS-T	/	−0.30 (−0.83,0.23)	0.30 (−0.14,0.75)	/	/	−0.60 (−1.29,0.10)	/	**0.909**	0.070	0.521	

Abbreviations: BARS-G, barnes akathisia scale global score; BARS-T, barnes akathisia scale total score; DTBZ, deutetrabenazine; ESS, epworth sleepiness scale; PBO, placebo; TBZ, tetrabenazine; UHDRS TMC, Unified Huntington’s Disease Rating Scale Total Maximal Chorea; VBZ, valbenazine. Bold values indicate statistically significant mean differences in pairwise comparisons or interventions ranking first in the surface under the cumulative ranking curve.

#### 3.3.2 UHDRS Total Motor score

Compared with placebo, valbenazine, deutetrabenazine, and tetrabenazine exhibited increased efficacy (MD = −4.30, 95% CrI = −6.81 to −1.78; MD = −4.00, 95% CrI = −6.47 to −1.53; MD = −3.30, 95% CrI = −3.91 to −2.69, respectively) (Table 2). The results of this analysis indicated that there were no statistically significant differences among the three interventions. The SUCRAs indicate a ranking of the three potential best treatments as valbenazine (0.781), deutetrabenazine (0.712) and tetrabenazine (0.506) (Figure 2B).

#### 3.3.3 The response to Clinical Global Impression of ChangeNomenclature

Compared with placebo, tetrabenazine, deutetrabenazine, and valbenazine exhibited increased efficacy (OR = 12.24, 95% CrI = 2.99 to 90.04; OR = 4.99, 95% CrI = 1.81 to 15.53; OR = 4.80, 95% CrI = 1.95 to 13.37, respectively) (Table 3). The results of this analysis indicated that there were no statistically significant differences among the three interventions. The SUCRAs indicate a ranking of the three potential best treatments as tetrabenazine (0.892), deutetrabenazine (0.564) and valbenazine (0.545) (Figure 2C).

**TABLE 3:** Fixed-effects model measures for interventions evaluated in the included studies have been represented by the odds ratio.

Outcomes measured	Network meta-analysis results (OR; 95% CI)						SUCRA			
	TBZ vs. PBO	DTBZ vs. PBO	VBZ vs. PBO	TBZ vs. DTBZ	TBZ vs. VBZ	DTBZ vs. VBZ	TBZ	DTBZ	VBZ	PBO
CGI-C response	**12.24 (2.99,90.04)**	**4.99 (1.81,15.53)**	**4.80 (1.95,13.37)**	2.48 (0.40,22.41)	2.57 (0.44,22.35)	1.04 (0.25,4.40)	**0.892**	0.564	0.545	0.000
PGI-C response	/	**4.33 (1.73,11.61)**	**2.82 (1.31,6.25)**	/	/	1.54 (0.45,5.29)	/	**0.876**	0.622	0.000
Withdrawals	3.84 (0.50,107.51)	0.40 (0.01,5.14)	0.98 (0.35,2.74)	10.77 (0.36,1019.07)	4.01 (0.39,126.93)	0.41 (0.01,6.46)	0.110	**0.800**	0.551	0.539
Withdrawals due to AEs	**3.65e6 (5.68,1.72e18)**	0.99 (0.02,37.99)	0.76 (0.17,3.13)	**3.80e6 (2.68,2.11e18)**	**4.91e6 (6.72,2.38e18)**	1.31 (0.02,64.54)	0.005	0.644	**0.735**	0.616
Dosage reduction	**33.86 (5.38,987.80)**	1.00 (0.16,6.19)	3.54 (0.96,17.53)	**35.97 (2.42,1549.60)**	9.86 (0.81,356.18)	0.28 (0.03,2.66)	0.014	**0.788**	0.375	0.824
AEs	**4.39 (1.32,16.06)**	1.21 (0.51,2.85)	1.90 (0.88,4.22)	3.65 (0.83,17.29)	2.31 (0.55,10.49)	0.64 (0.20,2.02)	0.060	**0.688**	0.382	0.870
SAE	**5.57e5 (3.53,1.46e18)**	0.99 (0.03,36.43)	0.40 (0.01,5.27)	**6.29e5 (1.61,1.62e18)**	**1.72e6 (5.28,3.7e18)**	2.60 (0.03,356.03)	0.009	0.605	**0.807**	0.579

Abbreviations: AEs, adverse events; CGI-C, clinical global impression of change; DTBZ, deutetrabenazine; PBO, placebo; PGI-C, patient global impression of change; SAE, serious adverse events; TBZ, tetrabenazine; VBZ, valbenazine.Bold values indicate statistically significant odds ratios in pairwise comparisons or interventions ranking first in the surface under the cumulative ranking curve.

#### 3.3.4 The response to Patient Global Impression of Change

Only two RCTs reported the response to Patient Global Impression of Change. Compared with placebo, deutetrabenazine and valbenazine exhibited increased efficacy (OR = 4.33, 95% CrI = 1.73 to 11.61; OR = 2.82, 95% CrI = 1.31 to 6.25, respectively) (Table 3). The results of this analysis indicated that there were no statistically significant differences between the two interventions. The SUCRAs indicate a ranking of the two potential best treatments as deutetrabenazine (0.876) and valbenazine (0.622) (Figure 2D).

### 3.4 Tolerability outcomes

Compared with placebo or with each other, none of the interventions showed a statistically significant effect on the incidence of overall withdrawals. However, tetrabenazine demonstrated a significantly higher incidence of withdrawals due to AEs compared to placebo (OR = 3.65e6, 95% CrI = 5.68 to 1.72e18), deutetrabenazine (OR = 3.80e6, 95% CrI = 2.68 to 2.11e18), and valbenazine (OR = 4.91e6, 95% CrI = 6.72 to 2.38e18) (Table 3). Moreover, tetrabenazine exhibited a greater risk of dosage reduction compared to placebo or deutetrabenazine (OR = 33.86, 95% CrI = 5.38 to 987.80; OR = 35.97, 95% CrI = 2.42–1549.60), though no statistically significant difference was observed versus valbenazine.

The SUCRAs indicate that tetrabenazine may be associated with a higher risk of three tolerability outcomes: overall withdrawals (0.110), withdrawals due to AEs (0.005), and dosage reduction (0.014). Except for placebo, deutetrabenazine ranked first in overall withdrawals (0.800) (Figure 2E) and dosage reduction (0.788), while valbenazine ranked first in withdrawals due to AEs (0.735) (Figure 2F).

### 3.5 Safety outcomes

Compared with placebo, only tetrabenazine among the three treatments was associated with an increased risk of both AEs and SAEs (OR = 4.39, 95% CrI = 1.32 to 16.06; OR = 5.57e5, 95% CrI = 3.53 to 1.46e18) (Table 3). While no statistical difference was observed among the three treatments for AEs, tetrabenazine showed significantly higher SAE risks compared to both deutetrabenazine and valbenazine (OR = 6.29e5, 95% CrI = 1.61 to 1.62e18; OR = 1.72e6, 95% CrI = 5.28 to 3.71e18).

The SUCRAs indicated that deutetrabenazine ranked first in AEs (0.688, except for placebo) (Figure 2G), while valbenazine ranked first in SAEs (0.807) (Figure 2H). Given the availability of comprehensive data, we conducted an analysis encompassing the ESS score, BARS global and total scores, somnolence, fatigue, falls, depression, insomnia, suicide, and suicidal ideation (Table 2; Supplementary Table S2). According to the ranking probabilities presented in Table 2; Supplementary Table S3, deutetrabenazine emerged as the optimal treatment among the three, showing the lowest risk of AEs related to the ESS, BARS global and total scores, somnolence, fatigue, falls, depression, and insomnia (0.835, 0.958, 0.909, 0.580, 0.469, 0.817, 0.846, and 0.654, respectively). Tetrabenazine is a better choice for reducing the risk of diarrhea (0.836). Valbenazine performed best in preventing suicide and suicidal ideation (0.683 and 0.748).

### 3.6 Radar chart

**FIGURE 3:**
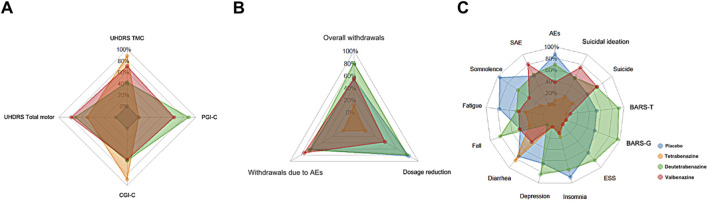
The radar chart for SUCRA values across three types of outcomes. Each color represents a different drug, and each axis label corresponds to a specific outcome. The larger the area covered by the color representing a drug on the radar chart, the more advantageous that drug is for that particular type of outcome. Abbreviations: UHDRS TMC, Unified Huntington’s Disease Rating Scale Total Maximal Chorea; CGI-C, Clinical Global Impression of Change; PGI-C, Patient Global Impression of Change; AEs, adverse events; SAE, Serious adverse events; ESS, Epworth Sleepiness Scale; BARS-G, Barnes Akathisia Scale global score; BARS-T, Barnes Akathisia Scale total score. **(A)** Efficacy indicators (SUCRA). **(B)** Tolerance indicators (SUCRA). **(C)** Safety indicators (SUCRA).

The three radar charts (Figure 3) depict overall drug and placebo performance in efficacy, safety, and tolerability, highlighting strengths and weaknesses across specific indicators. The three radar charts depict overall drug and placebo performance in efficacy, safety, and tolerability, highlighting strengths and weaknesses across specific indicators.

In terms of efficacy outcomes, the radar plots of all three active treatments completely encompassed the placebo group across all endpoints, though with minimal differences in radar plot area between them. Notably, tetrabenazine demonstrated advantages in UHDRS TMC and CGI-C scores despite having missing PGI-C data (recorded as 0, suggesting its true area may be larger), while valbenazine showed superiority in UHDRS Total Motor scores and deutetrabenazine outperformed in PGI-C assessments.

Regarding tolerability, deutetrabenazine and valbenazine exhibited substantially larger radar plot areas that completely covered tetrabenazine’s plot, clearly indicating tetrabenazine’s inferior tolerability profile. Specifically, deutetrabenazine performed better in overall withdrawals and dosage reductions, whereas valbenazine had fewer withdrawals due to adverse events.

For safety outcomes, deutetrabenazine displayed the largest radar plot area with more prominent vertices across most safety events, except for SAEs and suicidal ideation (where valbenazine was superior) and diarrhea (where tetrabenazine showed a distinct advantage). In contrast, tetrabenazine’s plot was the smallest and was nearly entirely overlapped by the other two drugs, with its only notable protrusions being in diarrhea and insomnia, confirming its generally poorer safety performance compared to deutetrabenazine and valbenazine.

### 3.7 Three-dimensional scatterplot

**FIGURE 4:**
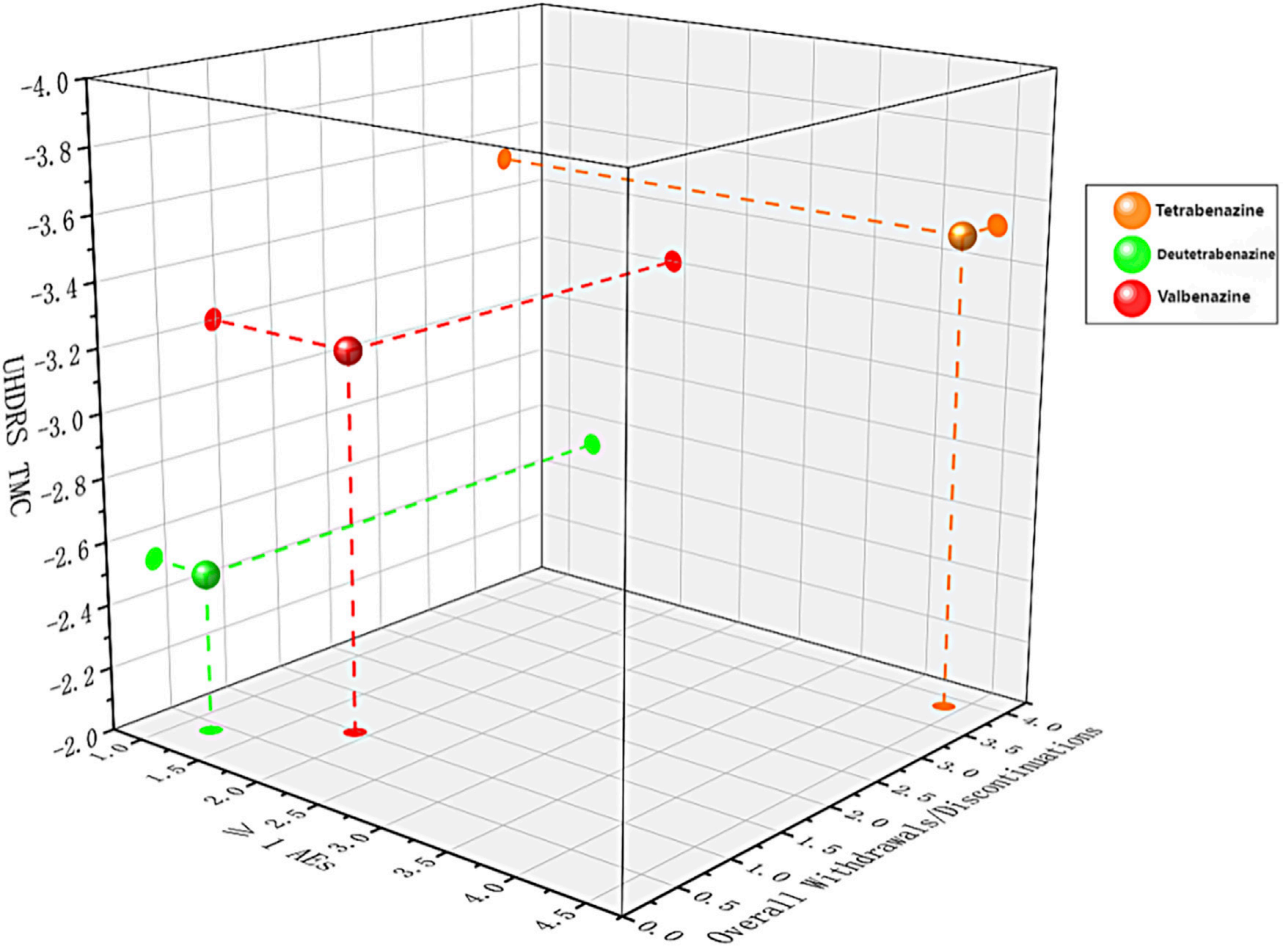
Three-dimension scatterplot of the network for three outcomes. Each plane shows two outcomes. Each color represents a group of treatments that belong to the same cluster. Treatments lying in the higher left corner are more effective and safer than the other treatments.

A three-dimensional scatter plot (Figure 4) combining primary efficacy outcomes, overall withdrawals, and ≥1 AEs was also generated. Tetrabenazine appeared higher up, indicating better efficacy; deutetrabenazine was positioned more to the left and closer, reflecting its advantage in safety and tolerability; while valbenazine, located toward the upper left relative to other treatments, demonstrated superior overall performance.

## 4 Discussion

This Bayesian NMA of RCTs found no statistically significant differences in efficacy among the three VMAT2 inhibitors for controlling chorea symptoms in Huntington’s disease. Tetrabenazine may be a more effective option for controlling chorea symptoms based on the SUCRA values of the UHDRS TMC score, but it has poor tolerability and a significantly higher risk of AEs and SAEs. Deutetrabenazine offers advantages in balancing dose adherence and mitigating most adverse reactions, such as depression, excessive daytime sleepiness, and akathisia, though its overall efficacy may be inferior to the other two VMAT2 inhibitors. In contrast, valbenazine demonstrates the best overall performance in terms of efficacy, tolerability, and safety, particularly in managing overall motor symptoms and high-risk events (SAEs and suicidality).

In terms of efficacy, VMAT2 inhibitors were associated with significant statistical differences in reduction of UHDRS TMC score, UHDRS Total motor score, CGI-C response and PGI-C response (except tetrabenazine for missing data) of HD compared to placebo in general. Among the three VMAT2 inhibitors, SUCRA values indicated that tetrabenazine provides the greatest benefit in improving chorea symptoms and global quality of life in HD, ranking first in UHDRS TMC score and CGI-C response. However, the potential superiority of tetrabenazine in UHDRS TMC score may be attributed to the higher mean baseline UHDRS TMC score observed in the studied HD patient population (Huntington Study Group, 2006). Although no literature currently confirms a direct relationship between tetrabenazine and HD disease severity, we hypothesize that this difference in baseline scores might contribute to its higher SUCRA ranking. Supporting this hypothesis, a nonrandomized, open-label switch cohort study by Samuel Frank et al. (ARC-HD) (Frank et al., 2022)—designed to assess long-term effects—reported a mean change in TMC score of −0.5, suggesting deutetrabenazine’s long-term efficacy is comparable to tetrabenazine’s. These findings imply that tetrabenazine’s efficacy advantage may be overstated; nevertheless, given the methodological limitations of the ARC-HD trial, we maintain that tetrabenazine retains superior overall efficacy among VMAT2 inhibitors.

To our knowledge, no clinical trials directly comparing valbenazine with other VMAT2 inhibitors for HD chorea have been completed and published. Based on our findings, valbenazine had higher SUCRA values than deutetrabenazine (but lower than tetrabenazine) in UHDRS TMC score, while it was superior to other VMAT2 inhibitors in UHDRS Total Motor Score. This suggests that valbenazine may be less effective than tetrabenazine in controlling chorea but more effective in improving overall motor symptoms in HD patients. It should be noted that no statistically significant differences were observed in the pairwise comparisons among the three VMAT2 inhibitors across the four efficacy outcomes, and the clinical relevance of these findings warrants further validation in additional RCTs.

In terms of tolerability, compared with placebo, only tetrabenazine was associated with a higher risk of withdrawals due to AEs and dosage reductions due to intolerance. Furthermore, tetrabenazine also demonstrated an increased risk compared with other VMAT2 inhibitors in terms of withdrawals due to AEs. SUCRAs further indicated that tetrabenazine performed worse than the other two VMAT2 inhibitors in overall withdrawals, withdrawals due to AEs, and dosage reductions due to intolerance. In the tolerability analysis, tetrabenazine was almost certainly the worst-performing among the VMAT2 inhibitors, a finding likely attributable to its frequent dosing regimen (three times daily) and higher peak dose (100 mg) (Huntington Study Group, 2006). The findings from the safety analysis suggest that this may be related to the higher risk of AEs and SAEs associated with tetrabenazine itself.

According to the SUCRA rankings, deutetrabenazine ranked highest in both overall withdrawals and dosage reductions due to intolerance, while valbenazine ranked highest in withdrawals due to AEs. It is worth noting that in the original valbenazine study (Stimming et al., 2023), seven cases of study discontinuation due to COVID-19 were included in the overall withdrawals, a factor not observed in other studies. This unique circumstance may have exaggerated the overall withdrawal risk of valbenazine compared to other VMAT2 inhibitors. Raw data also indicate that in other studies or the valbenazine placebo group, reasons for withdrawal were primarily objective factors such as AE occurrence or physician-assessed lack of efficacy, whereas the higher overall withdrawals for valbenazine were additionally influenced by participant withdrawals due to subjective factors, which were not observed in the valbenazine placebo group or the other two studies. Therefore, we conclude that valbenazine demonstrates better tolerability than deutetrabenazine with regard to withdrawals due to AEs, while deutetrabenazine shows improved outcomes in minimizing dosage reductions due to intolerance. However, these unique circumstances suggest that deutetrabenazine may not demonstrate a substantial advantage over valbenazine in overall tolerability. Notably, tetrabenazine consistently exhibited inferior tolerability compared to both other VMAT2 inhibitors across all evaluated outcomes.

We also conducted a safety analysis. Compared with placebo, only tetrabenazine was associated with higher incidences of AEs and SAEs. Additionally, it performed worse in reducing SAE risk compared with other VMAT2 inhibitors. This may be related to the fact that tetrabenazine’s metabolism is influenced by the cytochrome P450 2D6 (CYP2D6) enzyme, leading to significant interindividual variability in metabolic rates (Gaedigk, 2013; Wang et al., 2023). In contrast, deutetrabenazine, benefiting from the enhanced chemical bond stability conferred by deuteration (Russak and Bednarczyk, 2019; Gupta and Kaur, 2017), and valbenazine, which undergoes metabolism primarily via the cytochrome P450 2D6 (CYP3A4/5) enzyme with minimal genetic polymorphism, both possess longer half-lives (Grigoriadis et al., 2017). These characteristics significantly diminish their respective metabolic variability.

According to the SUCRA ranking, deutetrabenazine was associated with lower risks of common AEs than the other two VMAT2 inhibitors. Specifically, it is associated with lower risks of somnolence, fatigue, falls, depression, and insomnia, as well as in improving ESS score, BARS global and total scores. However, it demonstrated a significantly higher incidence of diarrhea compared to placebo or other VMAT2 inhibitors. Valbenazine was associated with a lower risk of SAEs. More important, it was associated with lower risks of suicidal ideation and suicide compared to the other two VMAT2 inhibitors Therefore, Valbenazine maybe a better choice for HD patients with psychiatric dysfunction. This may be attributed to its high selectivity and the single nature of its metabolites, which contributed to reducing off-target effects of the drug (Skor et al., 2017; Brar et al., 2022).

As with any network meta-analysis, there are some limitations which should be mentioned to appropriately interpret the results of the present study. The main limitations include the insufficient number of RCTs (n = 3, with only one trial per VMAT2 inhibitor) and the absence of direct drug comparisons, which precluded standard evaluations of publication bias and consistency, as well as CINeMA assessments for imprecision, heterogeneity, and incoherence. Furthermore, the limited sample size resulted in excessively wide credible intervals and extreme OR values for some binary outcomes, largely due to zero-event cells in the original studies, underscoring the need for cautious interpretation of certain safety and tolerability results. Furthermore, the UHDRS TMC scores in TETRA-HD (Huntington Study Group, 2006) are higher than those in the other two studies. Secondly, the sample size of the included RCTs is also small. A total of 299 HD patients were included in the present study and in the study of TETRA-HD (Huntington Study Group, 2006), only 84 patients were included. Furthermore, all the RCTs included were performed in the United States and Canada, with predominantly white patient populations. Therefore, the efficacy and safety of VMAT2 inhibitors in other ethnic groups of HD patients may need to be confirmed in further studies. Thirdly, the follow-up time is short. The overall treatment durations of all the RCTs included were 12 weeks. Therefore, the longterm efficacy and safety of VMAT2 inhibitors in HD patients cannot be fully explored.

## 5 Conclusion

In summary, this NMA suggests three VMAT2 inhibitors are effective in ameliorating chorea symptoms in patients with Huntington’s disease. Our findings also indicate that tetrabenazine is the most effective in controlling chorea, whereas valbenazine may be the optimal choice for patients with comorbid psychiatric symptoms. Furthermore, once daily of valbenazine may improve the patient’s adherence. Further study, particularly head-to-head, larger sample sizes, long follow-up time, randomized controlled trials are warranted to confirm our findings.

## Data Availability

The original contributions presented in the study are included in the article/Supplementary Material, further inquiries can be directed to the corresponding author.
