# Supplementary material for: Efficacy and safety of vesicular monoamine transporter 2 inhibitors for Huntington’s disease chorea based on network meta-analysis
Source: Front Pharmacol. 2025 Sep 24;16:1637577. doi: 10.3389/fphar.2025.1637577 (PMC12504331; doi:10.3389/fphar.2025.1637577)
Supplement: Supplementary file 1 [file Supplementaryfile1.docx]

**Supplementary Material**

**Supplementary Appendix S1.** Search Strategy used in our article.

**Supplementary Figure S1.** PRISMA flow chart.

**Supplementary Figure S2.** Trace plot and density plot in the primary outcomes.

**Supplementary Table S1.** Model fit details including the random effect (RE) compared and the fixed effect (FE) model.

**Supplementary Table S2.** Adverse effects for three treatments compared with placebo.

**Supplementary Table S3.** SUCRA values of three treatment modalities under eight adverse effects.

**Supplementary Table S4.** CINeMA Assessment in the primary outcomes.

**Appendix S1. Search** Strategy used in our article.

Complete Search algorithm used in MEDLINE

(Huntington OR "Huntington's Disease" OR "Huntington Disease" OR "Huntington Chorea" OR "Chorea, Huntington") AND (valbenazine OR deutetrabenazine OR "Austedo" OR tetrabenazine OR "Xenazine" OR "Nitoman" OR "vesicular monoamine transporter 2 inhibitor") AND (randomized OR randomised OR random OR randomly OR 'clinical trial')

Complete Search algorithm used in EMBASE

(Huntington OR "Huntingtons Disease" OR "Huntington Disease" OR "Huntington Chorea" OR "Chorea, Huntington") AND (valbenazine OR deutetrabenazine OR "Austedo" OR tetrabenazine OR "Xenazine" OR "Nitoman" OR "vesicular monoamine transporter 2 inhibitor") AND (randomized OR randomised OR random OR randomly OR 'clinical trial')

Complete Search algorithm used in the Cochrane Central Register of Controlled Trials

(Huntington OR "Huntington's Disease" OR "Huntington Disease" OR "Huntington Chorea" OR "Chorea, Huntington") AND (valbenazine OR deutetrabenazine OR "Austedo" OR tetrabenazine OR "Xenazine" OR "Nitoman" OR "vesicular monoamine transporter 2 inhibitor") AND (randomized OR randomised OR random OR randomly OR 'clinical trial')

**Figure S1.** PRISMA flow chart.


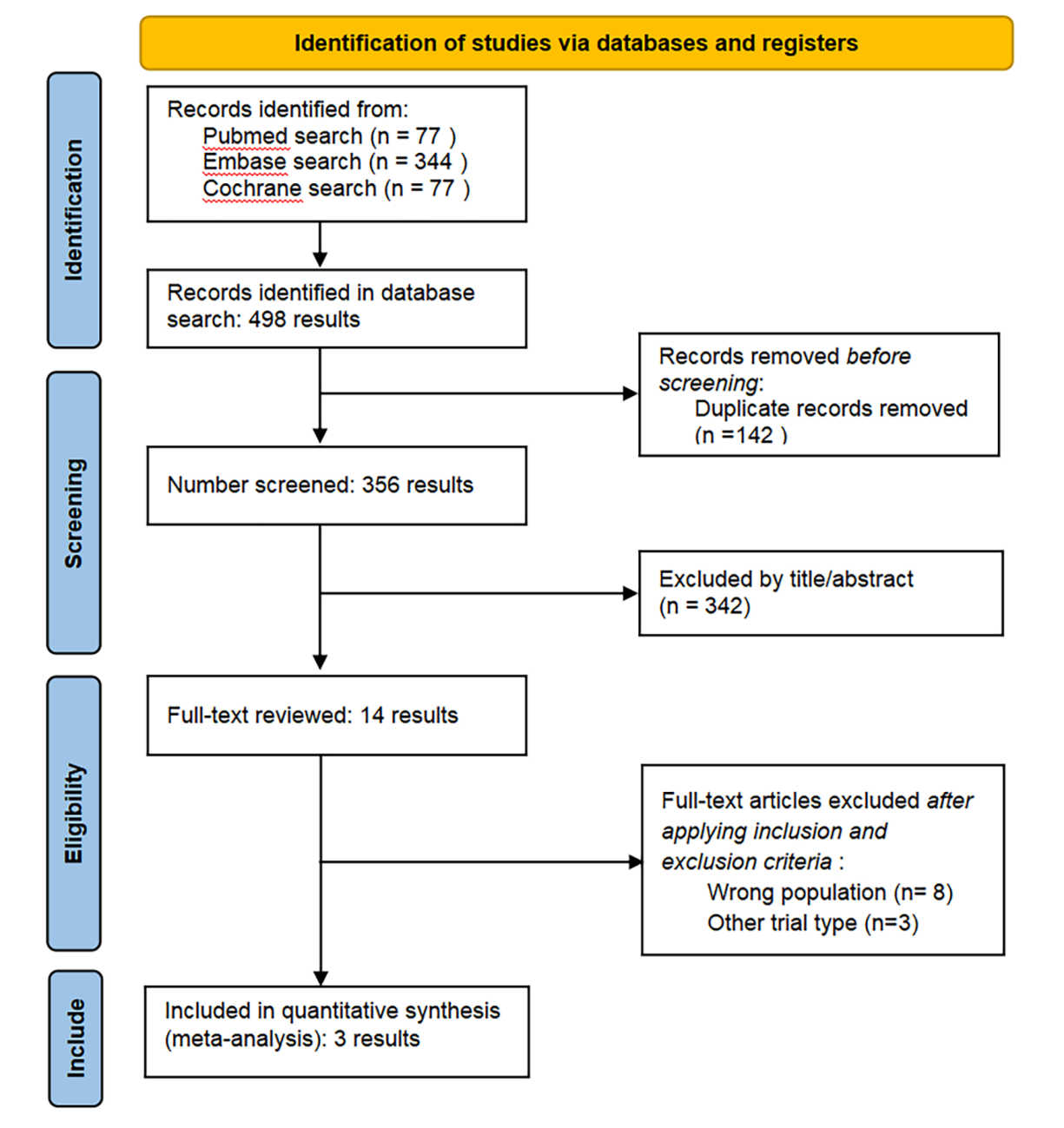


**Figure S2.** Trace plot and density plot in the primary outcomes.

1. Trace plot and density plot for UHDRS TMC score.


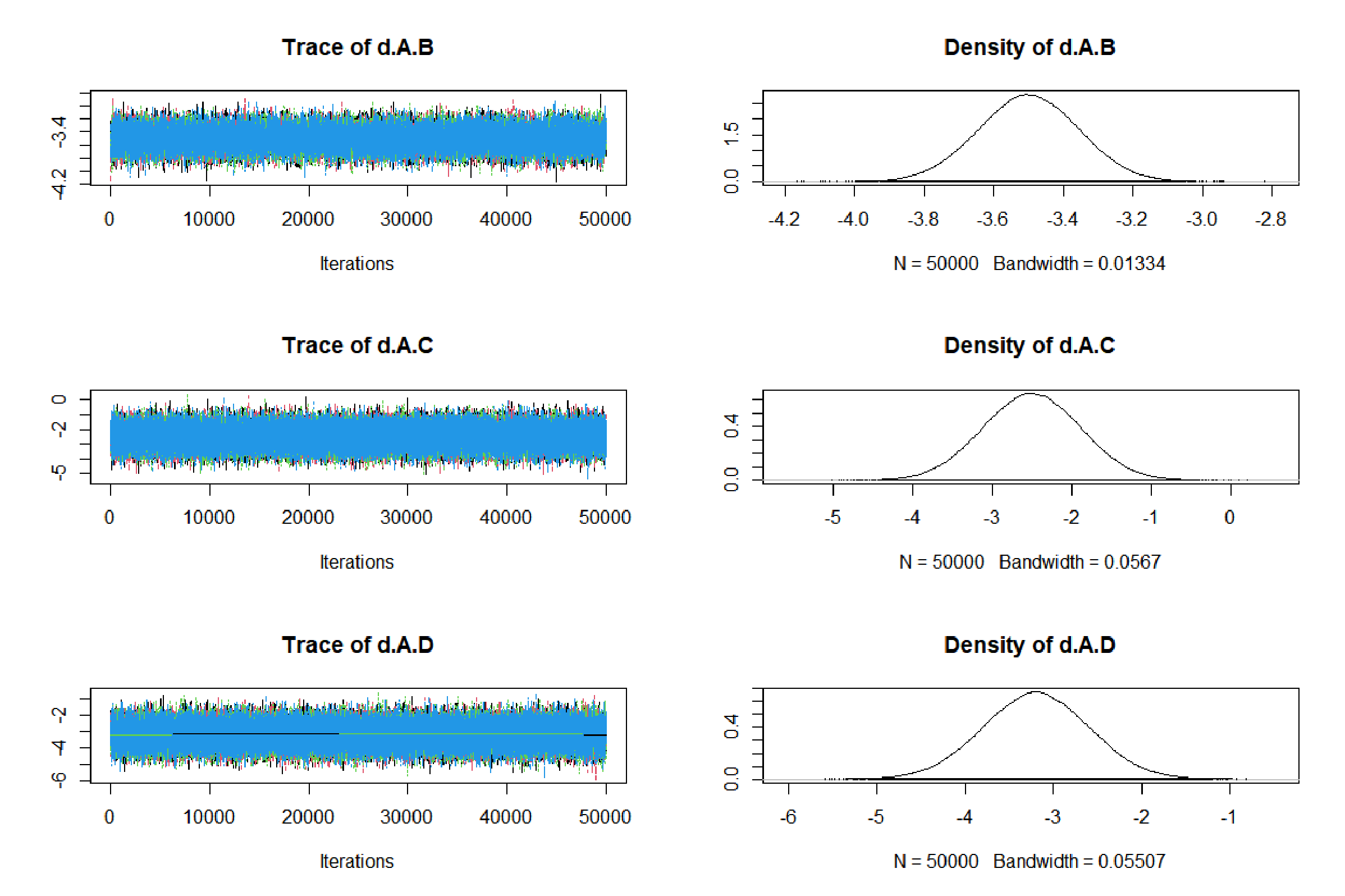


There are no specific patterns and the chains are entangled, it is considered that the convergence is good. A: Placebo; B: Tetrabenazine; C: Deutetrabenazine; D: Valbenazine.

1. Trace plot and density plot for UHDRS Total Motor score.


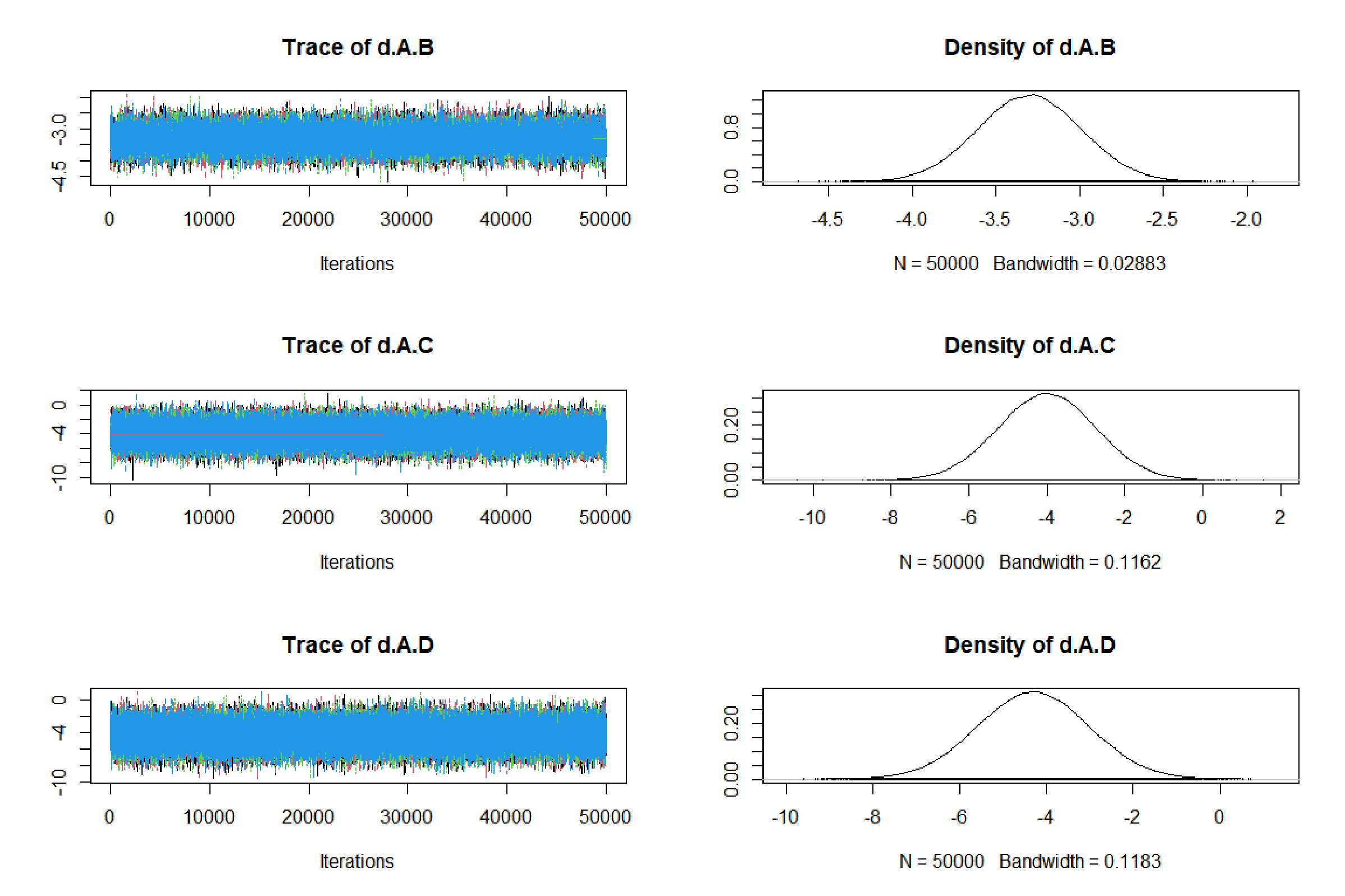


There are no specific patterns and the chains are entangled, it is considered that the convergence is good. A: Placebo; B: Tetrabenazine; C: Deutetrabenazine; D: Valbenazine.

1. Trace plot and density plot for the response to Clinical Global Impression of Change.


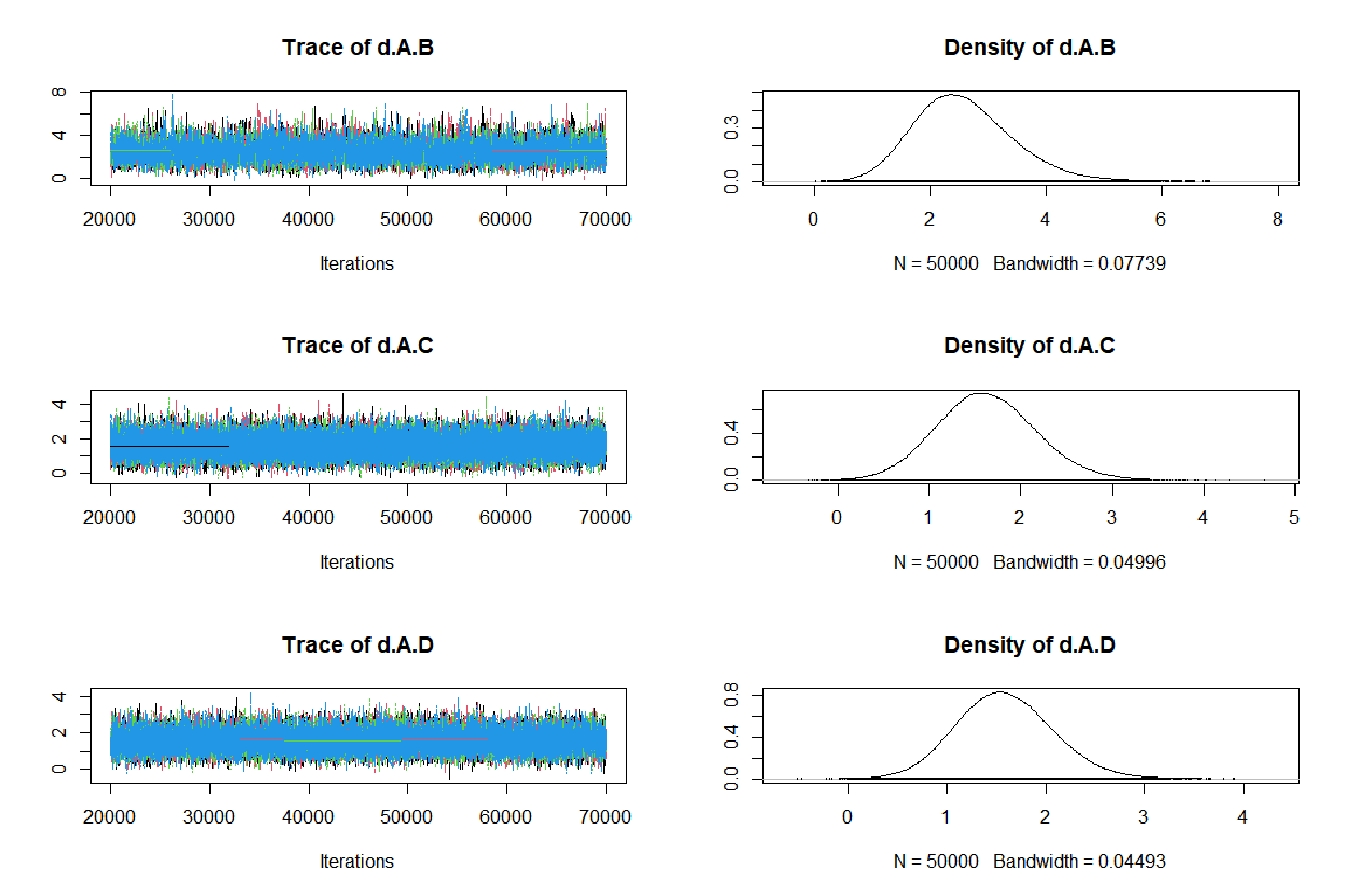


There are no specific patterns and the chains are entangled, it is considered that the convergence is good. A: Placebo; B: Tetrabenazine; C: Deutetrabenazine; D: Valbenazine.

1. Trace plot and density plot for the response to Patient Global Impression of Change.


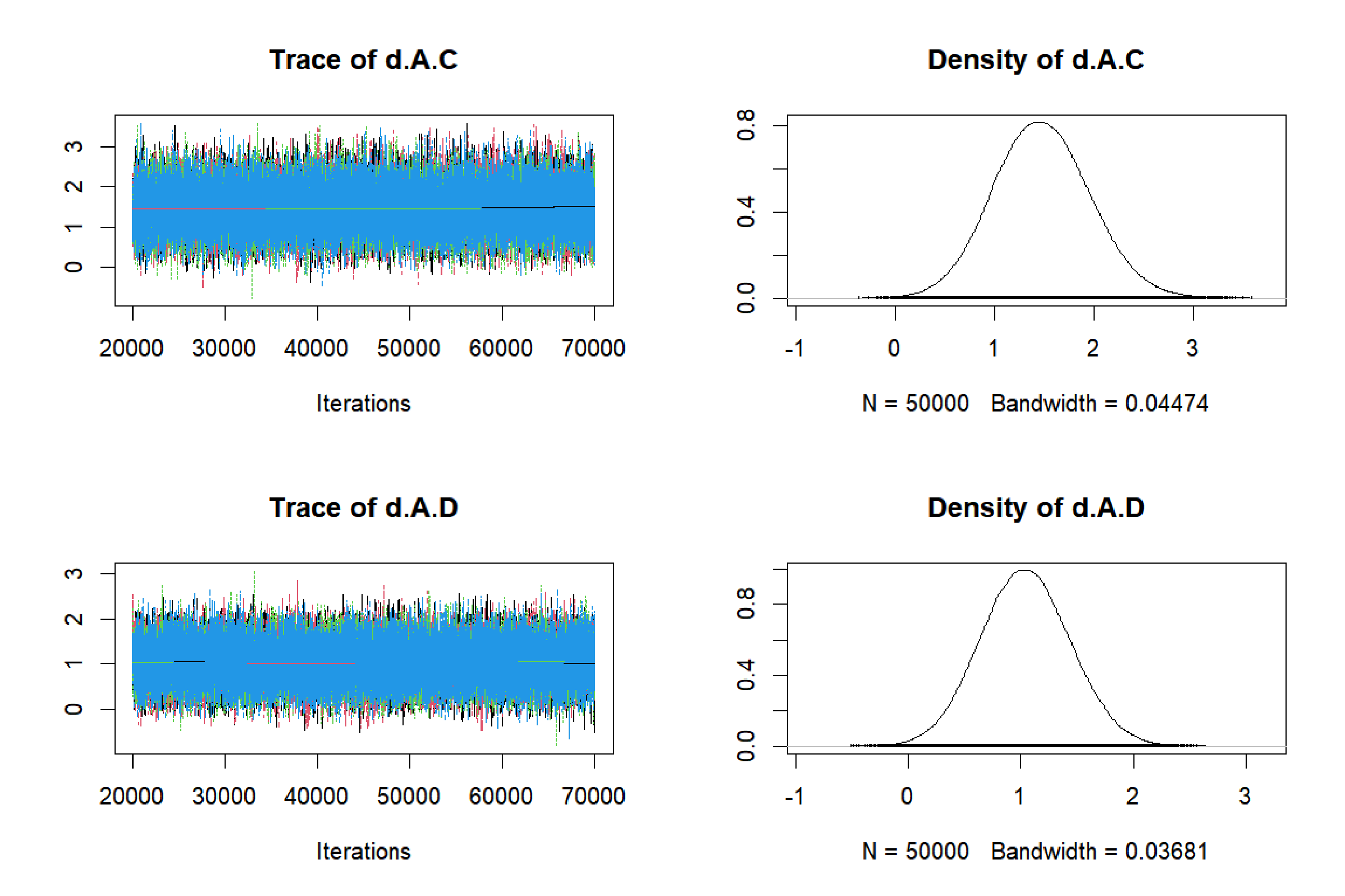


There are no specific patterns and the chains are entangled, it is considered that the convergence is good. A: Placebo; C: Deutetrabenazine; D: Valbenazine.

**Table S1.** Model fit details including the random effect (RE) compared and the fixed effect (FE) model.

|  | Deviance | unconstrained  data points | pD | DIC | ratio | I² |
| --- | --- | --- | --- | --- | --- | --- |
| UHDRS TMC RE | 6.00 | 6 | 6.00 | 12.00 | 1.00 | 17% |
| UHDRS TMC FE | 6.00 | 6 | 6.00 | 12.04 | 1.00 | 17% |
| UHDRS Total motor RE | 6.02 | 6 | 6.02 | 12.03 | 1.00 | 17% |
| UHDRS Total motor FE | 6.00 | 6 | 6.00 | 11.99 | 1.00 | 17% |
| ESS RE | 4.01 | 4 | 4.01 | 8.02 | 1.00 | 25% |
| ESS FR | 4.00 | 4 | 4.00 | 8.01 | 1.00 | 25% |
| BARS-G RE | 4.00 | 4 | 4.00 | 7.99 | 1.00 | 25% |
| BARS-G FE | 4.00 | 4 | 4.00 | 7.99 | 1.00 | 25% |
| BARS-T RE | 3.97 | 4 | 3.97 | 7.94 | 0.99 | 24% |
| BARS-T FE | 3.99 | 4 | 3.99 | 7.98 | 1.00 | 25% |
| CGI-C RE | 6.15 | 6 | 6.15 | 12.29 | 1.03 | 19% |
| CGI-C FE | 6.15 | 6 | 6.15 | 12.30 | 1.03 | 19% |
| PGI-C RE | 4.04 | 4 | 4.04 | 8.09 | 1.01 | 26% |
| PGI-C FE | 4.04 | 4 | 4.04 | 8.08 | 1.01 | 26% |
| AE RE | 6.09 | 6 | 6.09 | 12.18 | 1.02 | 18% |
| AE FE | 6.10 | 6 | 6.10 | 12.19 | 1.02 | 18% |
| SAE RE | 5.66 | 6 | 5.56 | 11.22 | 0.94 | 12% |
| SAE FE | 5.64 | 6 | 5.56 | 11.20 | 0.94 | 11% |
| Somnolence RE | 6.40 | 6 | 6.40 | 12.80 | 1.07 | 22% |
| Somnolence FE | 6.33 | 6 | 6.33 | 12.65 | 1.06 | 21% |
| Fatigue RE | 6.17 | 6 | 6.16 | 12.33 | 1.03 | 19% |
| Fatigue FE | 6.17 | 6 | 6.17 | 12.34 | 1.03 | 19% |
| Fall RE | 6.16 | 6 | 6.16 | 12.32 | 1.03 | 19% |
| Fall FE | 6.19 | 6 | 6.19 | 12.39 | 1.03 | 19% |
| Diarrhea RE | 5.39 | 6 | 5.32 | 10.71 | 0.90 | 7% |
| Diarrhea FE | 5.41 | 6 | 5.34 | 10.75 | 0.90 | 8% |
| Depression RE | 3.24 | 4 | 3.17 | 6.41 | 0.81 | 7% |
| Depression FE | 3.25 | 4 | 3.17 | 6.42 | 0.81 | 8% |
| Insomnia RE | 3.22 | 4 | 3.15 | 6.37 | 0.80 | 7% |
| Insomnia FE | 3.22 | 4 | 3.16 | 6.37 | 0.80 | 7% |
| Withdrawals RE | 6.35 | 6 | 6.35 | 12.69 | 1.06 | 21% |
| Withdrawals FE | 6.39 | 6 | 6.39 | 12.79 | 1.07 | 22% |
| Withdrawals due to AEs RE | 5.51 | 6 | 5.41 | 10.92 | 0.92 | 9% |
| Withdrawals due to AEs FE | 5.48 | 6 | 5.40 | 10.88 | 0.91 | 9% |
| Dosage reduction RE | 6.30 | 6 | 6.30 | 12.60 | 1.05 | 21% |
| Dosage reduction FE | 6.38 | 6 | 6.38 | 12.77 | 1.06 | 22% |

**Table S2.** Adverse effects for three treatments compared with placebo.

| Treatment | Somnolence | Fatigue | Fall | Diarrhea | Depression | Insomnia | Suicide | Suicidal ideation |
| --- | --- | --- | --- | --- | --- | --- | --- | --- |
| Tetrabenazine | **18.82**  **(2.90,452.30)** | 1.94  (0.59,7.70) | 1.36  (0.38,5.49) | 0.74  (0.14,4.34) | **8.75e6**  **(9.59,2.22e23)** | **5.13e9**  **(31.94,1.49e29)** | 15.27  (0.10,6.81e4) | 16.18  (0.09,6.83e4) |
| Deutetrabenazine | 2.99  (0.56,24.81) | 1.56  (0.23,13.42) | 0.44  (0.05,2.52) | **1.07e7**  **(7.98,8.26e22)** | 2.99  (0.56,24.81) | 1.63  (0.24,14.66) | 0.05  (4.86e-08,2362.00) | 0.92  (0.03,28.53) |
| Valbenazine | **6.53**  **(1.52,47.97)** | 1.58  (0.52,5.02) | 0.98  (0.33,2.90) | 3.91  (0.41,114.70) | / | **1.44e11**  **(11.35,2.84e35)** | 0.04  (4.42e-08,1916.00) | 0.04  (3.99e-08,1916.00) |

**Table S3.** SUCRA values of three treatment modalities under eight adverse effects.

| Treatment | Somnolence | Fatigue | Fall | Diarrhea | Depression | Insomnia | Suicide | Suicidal ideation |
| --- | --- | --- | --- | --- | --- | --- | --- | --- |
| Placebo | 0.965 | 0.774 | 0.448 | 0.744 | 0.654 | 0.897 | 0.473 | 0.535 |
| Tetrabenazine | 0.106 | 0.322 | 0.274 | **0.836** | 0.000 | 0.181 | 0.168 | 0.173 |
| Deutetrabenazine | **0.580** | **0.469** | **0.817** | 0.009 | **0.846** | **0.767** | 0.677 | 0.544 |
| Valbenazine | 0.349 | 0.435 | 0.461 | 0.411 | / | 0.115 | **0.683** | **0.748** |

**Table S4.** CINeMA Assessment in the primary outcomes.

1. CINeMA Assessment Results for UHDRS TMC score.

| Comparison | Number of  studies | Within-study bias | Reporting  bias | Indirectness | Imprecision | Heterogeneity | Incoherence | Confidence  rating | Reason(s) for downgrading |
| --- | --- | --- | --- | --- | --- | --- | --- | --- | --- |
| Mixed evidence | | | | | | | | | |
| DTBZ:PBO | 1 | No concerns | Low risk | No concerns | -- | -- | -- | High | -- |
| TBZ:PBO | 1 | No concerns | Low risk | No concerns | -- | -- | -- | High | -- |
| VBZ:PBO | 1 | No concerns | Some concerns | No concerns | -- | -- | -- | Moderate | ["Reporting bias"] |
| Indirected evidence | | | | | | | | | |
| DTBZ:TBZ | 0 | No concerns | Low risk | No concerns | -- | -- | -- | High | -- |
| DTBZ:VBZ | 0 | No concerns | Some concerns | No concerns | -- | -- | -- | Moderate | ["Reporting bias"] |
| TBZ:VBZ | 0 | No concerns | Some concerns | No concerns | -- | -- | -- | Moderate | ["Reporting bias"] |

Confidence in the evidence was generally high for most direct comparisons, though some concerns remained for valbenazine due to limited data availability. The CINeMA tool could not formally evaluate imprecision, heterogeneity, or incoherence due to network sparsity and the limited number of studies, which precluded meaningful statistical assessment for these domains.

1. CINeMA Assessment Results for UHDRS Total Motor score.

| Comparison | Number of  studies | Within-study bias | Reporting  bias | Indirectness | Imprecision | Heterogeneity | Incoherence | Confidence  rating | Reason(s) for downgrading |
| --- | --- | --- | --- | --- | --- | --- | --- | --- | --- |
| Mixed evidence | | | | | | | | | |
| DTBZ:PBO | 1 | No concerns | Low risk | No concerns | -- | -- | -- | High | -- |
| TBZ:PBO | 1 | No concerns | Low risk | No concerns | -- | -- | -- | High | -- |
| VBZ:PBO | 1 | No concerns | Some concerns | No concerns | -- | -- | -- | Moderate | ["Reporting bias"] |
| Indirected evidence | | | | | | | | | |
| DTBZ:TBZ | 0 | No concerns | Low risk | No concerns | -- | -- | -- | High | -- |
| DTBZ:VBZ | 0 | No concerns | Some concerns | No concerns | -- | -- | -- | Moderate | ["Reporting bias"] |
| TBZ:VBZ | 0 | No concerns | Some concerns | No concerns | -- | -- | -- | Moderate | ["Reporting bias"] |

Confidence in the evidence was generally high for most direct comparisons, though some concerns remained for valbenazine due to limited data availability. The CINeMA tool could not formally evaluate imprecision, heterogeneity, or incoherence due to network sparsity and the limited number of studies, which precluded meaningful statistical assessment for these domains.

1. CINeMA Assessment Results for response to Patient Global Impression of Change.

| Comparison | Number of  studies | Within-study bias | Reporting  bias | Indirectness | Imprecision | Heterogeneity | Incoherence | Confidence  rating | Reason(s) for downgrading |
| --- | --- | --- | --- | --- | --- | --- | --- | --- | --- |
| Mixed evidence | | | | | | | | | |
| DTBZ:PBO | 1 | No concerns | Low risk | No concerns | -- | -- | -- | High | -- |
| TBZ:PBO | 1 | No concerns | Low risk | No concerns | -- | -- | -- | High | -- |
| VBZ:PBO | 1 | No concerns | Some concerns | No concerns | -- | -- | -- | Moderate | ["Reporting bias"] |
| Indirected evidence | | | | | | | | | |
| DTBZ:TBZ | 0 | No concerns | Low risk | No concerns | -- | -- | -- | High | -- |
| DTBZ:VBZ | 0 | No concerns | Some concerns | No concerns | -- | -- | -- | Moderate | ["Reporting bias"] |
| TBZ:VBZ | 0 | No concerns | Some concerns | No concerns | -- | -- | -- | Moderate | ["Reporting bias"] |

Confidence in the evidence was generally high for most direct comparisons, though some concerns remained for valbenazine due to limited data availability. The CINeMA tool could not formally evaluate imprecision, heterogeneity, or incoherence due to network sparsity and the limited number of studies, which precluded meaningful statistical assessment for these domains.

1. CINeMA Assessment Results for response to Patient Global Impression of Change.

| Comparison | Number of  studies | Within-study bias | Reporting  bias | Indirectness | Imprecision | Heterogeneity | Incoherence | Confidence  rating | Reason(s) for downgrading |
| --- | --- | --- | --- | --- | --- | --- | --- | --- | --- |
| Mixed evidence | | | | | | | | | |
| DTBZ:PBO | 1 | No concerns | Low risk | No concerns | -- | -- | -- | High | -- |
| VBZ:PBO | 1 | No concerns | Some concerns | No concerns | -- | -- | -- | Moderate | ["Reporting bias"] |
| Indirected evidence | | | | | | | | | |
| DTBZ:VBZ | 0 | No concerns | Some concerns | No concerns | -- | -- | -- | Moderate | ["Reporting bias"] |

Confidence in the evidence ranged from moderate to high for direct comparisons, though some concerns remained for valbenazine due to limited data availability. The CINeMA tool could not formally evaluate imprecision, heterogeneity, or incoherence due to network sparsity and the limited number of studies, which precluded meaningful statistical assessment for these domains.
